# Dync1li1 is required for the survival of mammalian cochlear hair cells by regulating the transportation of autophagosomes

**DOI:** 10.1371/journal.pgen.1010232

**Published:** 2022-06-21

**Authors:** Yuan Zhang, Shasha Zhang, Han Zhou, Xiangyu Ma, Leilei Wu, Mengyao Tian, Siyu Li, Xiaoyun Qian, Xia Gao, Renjie Chai

**Affiliations:** 1 Department of Otolaryngology Head and Neck Surgery, Affiliated Drum Tower Hospital of Nanjing University Medical School, Jiangsu Provincial Key Medical Discipline (Laboratory), Nanjing, China; 2 Research Institute of Otolaryngology, Nanjing, China; 3 State Key Laboratory of Bioelectronics, Department of Otolaryngology Head and Neck Surgery, Zhongda Hospital, School of Life Sciences and Technology, Advanced Institute for Life and Health, Jiangsu Province High-Tech Key Laboratory for Bio-Medical Research, Southeast University, Nanjing, China; 4 Department of Otolaryngology Head and Neck Surgery, Sichuan Provincial People’s Hospital, University of Electronic Science and Technology of China, Chengdu, China; 5 Co-Innovation Center of Neuroregeneration, Nantong University, Nantong, China; 6 Institute for Stem Cell and Regeneration, Chinese Academy of Science, Beijing, China; 7 Beijing Key Laboratory of Neural Regeneration and Repair, Capital Medical University, Beijing, China; Creighton University School of Medicine, UNITED STATES

## Abstract

Dync1li1, a subunit of cytoplasmic dynein 1, is reported to play important roles in intracellular retrograde transport in many tissues. However, the roles of Dync1li1 in the mammalian cochlea remain uninvestigated. Here we first studied the expression pattern of Dync1li1 in the mouse cochlea and found that Dync1li1 is highly expressed in hair cells (HCs) in both neonatal and adult mice cochlea. Next, we used *Dync1li1* knockout (KO) mice to investigate its effects on hearing and found that deletion of Dync1li1 leads to early onset of progressive HC loss via apoptosis and to subsequent hearing loss. Further studies revealed that loss of Dync1li1 destabilizes dynein and alters the normal function of dynein. In addition, *Dync1li1* KO results in a thinner Golgi apparatus and the accumulation of LC3+ autophagic vacuoles, which triggers HC apoptosis. We also knocked down *Dync1li1* in the OC1 cells and found that the number of autophagosomes were significantly increased while the number of autolysosomes were decreased, which suggested that *Dync1li1* knockdown leads to impaired transportation of autophagosomes to lysosomes and therefore the accumulation of autophagosomes results in HC apoptosis. Our findings demonstrate that Dync1li1 plays important roles in HC survival through the regulation of autophagosome transportation.

## Author summary

Hearing loss is one of the most common sensorial disorders globally. The main reason of hearing loss is the irreversible loss or malfunction of cochlear hair cells. Identifying new hearing loss-related genes and investigating their roles and mechanisms in HC survival are important for the prevention and treatment of hereditary hearing loss. Cytoplasmic dynein 1 is reported to play important roles in in ciliogenesis and protein transport in the mouse photoreceptors. Here, we described the expression pattern of Dyncili1 (a subunit of cytoplasmic dynein 1) in the mouse cochlea and used knockout mice to investigate its specific role in the hair cell of cochlea.

## Introduction

Hearing loss is one of the most common sensorial disorders globally, and it leads to reduced quality of daily life and to huge economic costs [1]. Genetic factors, aging, intense noise exposure, and aminoglycoside treatment can cause hearing loss, mainly through the irreversible loss or malfunction of cochlear hair cells (HCs) [2]. Genetic factors causing hereditary hearing loss remain to be identified. Therefore, identifying new hearing loss-related genes and investigating their roles and mechanisms in HC survival are important for the prevention and treatment of hereditary hearing loss.

Cytoplasmic Dynein1 (hereafter referred to as dynein), responsible for intracellular retrograde transport, is a multi-subunit complex that consists of two copies of the 530 kDa dynein heavy chain (DHC), two 74 kDa dynein intermediate chains (DICs), two 53–57 kDa dynein light intermediate chains (DLICs), and three 8–21 kDa light chains (DLCs) [3,4]. The DHC harbors the ATPase and is responsible for force production. The DIC plays a key scaffolding role in the complex and is involved in communicating with other protein complexes to regulate dynein activity [3,5]. It is reported that the DLC can act as an adapter to link various proteins to the dynein motor complex [6–8]. The DLIC is an essential subunit of dynein and is highly conserved in different eukaryotic cells [9]. In vertebrates, there are two DLIC genes, *Dync1li1* and *Dync1li2*, while there is only one DLIC gene in lower eukaryotes [10,11]. The C-terminus of the DLIC contains two conserved regions with helical propensity, and the N-terminal GTPase-like domain is also conserved and tightly binds to the DHC [7,12,13]. Knockdown (KD) or knockout (KO) of Dync1li1 in vitro leads to mitotic defects, fragmentation of the Golgi apparatus, and abnormal intracellular vesicle transport [14–17], and depletion of Dync1li1 in Drosophila cells and Aspergillus nidulans leads to destabilization of the DHC and DIC [14,18]. Mice with a point mutation in Dync1li1 show increased length of dendrites in cortical neurons and an increased number of dendrite branches in dorsal root ganglia neurons [19]. In the retina, Dync1li1 regulates the transportation of membrane proteins of rod outer segment from the Golgi to the base of the connecting cilium, thus regulating the formation of primary cilia [20]. Considering that the retina and the cochlea are both sensorial organs and might share some common mechanisms, we hypothesize that Dync1li1 plays important roles in the cochlea.

Autophagy is a highly conserved homeostatic process that eliminates defective organelles and misfolded proteins [21–23]. Several studies have suggested that there is a close relationship between autophagy and hearing loss in animal models [24–26]. Autophagic flux includes autophagosome formation, transportation and fusion with the lysosome, and finally maturation to form autolysosomes (both autophagosome and autolysosomes are called autophagic vacuoles) [27]. Disruption of autophagic flux will prevent autophagosomes from being cleared from the cell, thus resulting in increased cellular stress and ultimately leading to cell death and subsequent neurodegenerative disorders [28–30]. Current research on autophagy in the auditory system is limited, and most such studies have focused on sensorineural hearing loss caused by exogenous HC damage [31].

It has been widely reported that there is a close relationship between dynein and autophagy [32]. When autophagy is induced due to nutrient starvation *in vitro*, DLCs help to release autophagic regulators from the dynein complex and thus initiate autophagosome nucleation [33,34]. In the brain, the impairment of dynein-driven autophagosome motility causes autophagosomes to accumulate in neurites and synaptic termini, and this indicates the importance of dynein and autophagy in the clearance of aggregate-prone proteins in preventing neurodegenerative diseases [35–39]. However, the relationships between dynein and autophagy in the inner ear remain to be investigated.

To understand the role of Dync1li1 in cochlear HCs and to elucidate the relationship between dynein and autophagy in the inner ear, we determined the expression pattern of Dync1li1 in the inner ear and investigated its role and mechanism in hearing function by using *Dync1li1* KO mice [20]. We found that Dync1li1 is highly expressed in HCs in both neonatal and adult mice and that *Dync1li1* KO leads to progressive HC loss via apoptosis and subsequently leads to hearing loss. We also found that deletion of *Dync1li1* resulted in a reduced number of Golgi lamellae and the accumulation of autophagosomes both *in vitro* and *in vivo*, which suggested that deletion of *Dync1li1* leads to HC apoptosis due to impaired transportation of autophagosomes. Overall, we provide evidence that Dync1li1 plays important roles in HC survival by regulating autophagosome transportation and Golgi-related vesicle trafficking.

## Materials and methods

### Ethics statement

Animals were maintained following the Rutgers University Institutional Animal Use and Care Committee (Protocol 201702497), National Institutes of Health guidelines, and the policies of the Expert Committee for the Approval of Projects of Experiments on Animals of the Academy of Sciences of the Czech Republic (Protocol 43/2015). These regulatory bodies approved all experimental procedures involving the animals.

All animal procedures were performed according to the protocols that were approved by the Animal Experimental Ethical Inspection Form of Southeast University (No.20210302028). All animal procedures were consistent with the National Institute of Health’s Guide for the Care and Use of Laboratory Animals.

### Animals

*Dync1li1* KO mice were a gift from Prof. Wufan Tao (Fudan University, Shanghai, China) [20]. LC3-GFP reporter mice of both sexes in the C57BL/6JNju background (Stock D000244, Nanjing Biomedical Research Institute of Nanjing University) were used in the experiments.

### Genotyping PCR

LC3-GFP reporter mice were genotyped by using genomic DNA from tail tips. Tail tips were digested by adding 180 μl 50 mM NaOH, incubating at 98°C for 1 h, and adding 20 μl 1M Tris-HCl pH 7.0. The genotyping primers were used as follows: wild type (F) 5′-TGA GCG AGC TCA AGA TAA TCA GGT-3′; wild type (R) 5′-GTT AGC ATT GAG CTG CAA GCG CCG TCT-3′; mutant (F): 5′-TCC TGC TGG AGT TCG TGA CCG-3′; mutant (R): 5′-TTG CGA ATT CTC AGC CGT CTT CAT CTC TCT CGC-3′. The PCR system for genotyping is as follows: genomic DNA 3 μl, primer of each 0.5 μl, 2× PCR mix (Vazyme, P112-03) 10 μl, and add H2O up to a total volume of 20 μl. The conditions of PCR were an initial denaturing step of 3 min at 94°C followed by 35 cycles of 35 s denaturation at 94°C, 30 s annealing at 60°C, and 40 s extension at 72°C. Genotyping of Dync1li1 KO mice was performed according to the previous report [20]. The genotyping primers of Dync1li1 were used as follows: wild type (F) 5′- GGA AGA TGT GAC AAG ACA GAC ACG -3′; wild type (R) 5′-TGG CTC AGT GGT AAA GGT CC -3′; mutant (F): 5′- GGA AGA TGT GAC AAG ACA GAC ACG -3′; mutant (R): 5′- TCA GGA AAA GCA CTG GCT G -3′.

### Auditory brainstem response (ABR) test

The mice were injected I.P. with 0.01 g/ml pentobarbital sodium (100 mg/kg body weight) to achieve deep anesthesia, and the closed-field ABR test were measured for thresholds is previously described [40]. The ABR test was performed in a soundproof room, and 3 fine needle-like electrodes were inserted at the cranial vertex, underneath the ear, and at the back near the tail of the mice. The frequency of ABR test are 4 kHz, 8 kHz, 12 kHz, 16 kHz, 24 kHz, and 32 kHz. The hearing thresholds were determined by decreasing the sound intensities from 90 dB in 20 dB steps until the lowest sound intensity of the first wave could be identified. All test was measured by TDT System III workstation running SigGen32 software (Tucker-Davis Technologies). The data were analyzed by using GraphPad Prism 7 software.

### Immunostaining and image acquisition

For P0–P7 neonatal mice, the cochlea was dissected with sharp forceps (WPI) in cold HBSS and the tissue fixed in 4% paraformaldehyde (PFA) for 1 h at room temperature (RT). When mice older than P7, the temporal bone was fixed in 4% PFA for 1 h, decalcified in 0.5 M EDTA solution for 1–3 days (it depends on the mice age) at RT, and then dissected in HBSS. The sample was washed by PBS and then blocked with blocking medium (5% donkey serum, 0.5% Triton X100, 0.02% sodium azide, and 1% bovine serum albumin in pH 7.4 PBS) for 1 h at RT. And then incubated with primary antibodies diluted in PBT1 medium (2.5% donkey serum, 0.1% Triton X100, 0.02% sodium azide, and 1% bovine serum albumin in pH 7.4 PBS) at 4°C overnight. The sample was then washed with 0.1% Triton X100 in pH 7.4 PBS for three times and incubated with fluorescence-conjugated secondary antibody (Invitrogen) or phalloidin (Invitrogen), both diluted 1:400 in PBT2 medium (0.1% Triton X100 and 1% bovine serum albumin in pH 7.4 PBS), for 1 h at RT. The sample was mounted in antifade fluorescence mounting medium (DAKO) after washing with 0.1% Triton X100 in pH 7.4 PBS for three times. The primary antibodies were anti-Myo7a (rabbit anti-myo7a; Proteus Bioscience, #25–6790; 1:1000 dilution in PBT1), phalloidin (Invitrogen, A34055), DAPI (Solarbio, C0060), anti-Dync1li1 (Abcam, ab157468, 1:400 dilution in PBT1), anti-Ctbp2 (BD Biosciences, #612044, 1:400 dilution in PBT1), anti-PSD95 (Millipore, #MAB1596, 1:400 dilution in PBT1), anti-Rab7 (abcam, ab137029, 1:400 dilution in PBT1). A TUNEL kit (Roche, 11684817910) was used to detect apoptotic cells according to the instructions. For image acquisition, all samples were scanned by Zeiss microscope (LSM 710) with the same hardware settings to enable direct comparison between treatment conditions. Since synapses are not always on the same layer, we performed Z projection with ImageJ software to capture the Ctbp2 (presynaptic marker) and PSD95 (postsynaptic marker) staining images in S3 Fig. HEI-OC1 cell immunohistochemistry protocol was the same as above.

### Scanning electron microscopy (SEM) and transmission electron microscopy (TEM)

As previously described [41], Temporal bones were collected and immediately fixed in 2.5% glutaraldehyde (Sigma-Aldrich, G5882) for 24 h and then in 1% OsO4 (Beijing Zhongxingbairui Technology) for 2h, then dehydrated in ethanol, dried, and then coated with gold. For SEM, samples were mounted on stubs, and sputter coated with gold (Cressington, 108). A scanning electron microscope (FEI Quanta 200) operating at 10 kV was used to take images of the hair bundles. For TEM, the sample further penetrated with graded propylene oxide (Macklin Biochemical, P816084) series and gradually polymerized in araldite. The ultrathin sections made by Leica powertone (Leica, Em UC6) were post-stained with uranyl acetate and lead citrate in turn, and examined by transmission electron microscope (Hitachi, H-7650). All pictures were taken by the electron microscope room of Nanjing Agricultural University.

### Real-time quantitative PCR

Samples were dissected to extract total RNA with Trizol reagent (Life, 15596–018) as previously described [41]. Reverse transcription of RNA into cDNA by RevertAid First Strand cDNA Synthesis Kit (Life, K1622), and real-time quantitative PCR (real-time qPCR) was performed by using the FastStart Universal SYBR Green Master (ROX) kit (Roche, 17747200) on Real-Time PCR System (Thermo Fisher Scientific) to quantify the levels of gene expression. The condition of qPCR was as follows: an initial denaturing step: 15 s at 95°C, then followed by 38 cycles of denaturation step:15 s at 95°C, then followed by annealing step: 60 s at 60°C, and 20 s extension at 72°C. Gapdh was used as the reference gene and used to normalize the mRNA expression data. Using the comparative cycle threshold (ΔΔCt) method to calculate the result. The primers of qPCR are shown in S1 Table.

### Western blotting

The basilar membranes of neonatal mice (P0–P7) and the temporal bones of mice older than P7 were dissected, and homogenized in ice-cold RIPA lysis buffer (Beyotime, P0013B) by using tissue homogenizer (Shanghai Jingxin Industrial Development Co., Ltd., JXFSTPRP -48). After centrifuged at 12,000 g for 15 min at 4°C, the supernatant was added with 5X SDS loading buffer (Beyotime, P0015L), separated by 10% or 15% SDS-PAGE, and transferred to an immobilon PVDF membrane (ISEQ00010, Millipore). After blocking with 5% non-fat dried milk in 0.1% PBS-Tween20 for 1 h at RT, the PVDF membrane was incubated with the primary antibody at 4°C overnight. After washing with 0.1% PBS-Tween20 for 5 times per 6 min, the membrane was incubated with HRP-conjugated second antibody (goat anti mouse HRP, M21OO1, goat anti rabbit HRP, M21OO2; ABMART) for 1hour at RT. Signals were detected with the West Femto Trial Kit (Product #34094; Thermo Scientific) on a FluorChem M system (FM0477; ProteinSimple). The primary antibodies were anti-Dync1li1 (abcam, ab157468), anti-Dync1li2 (Proteintech,18885-1-AP), anti- Dync1i1/2 (DIC) (Millipore, MAB1618), anti-Dynll1 (DLC) (abcam, ab51603), anti-Rab7 (abcam, ab137029), anti-LC3 (CST, #4108), anti-Calnexin (santa cruz, sc-70481), anti-P-eIF2α (CST, 3597S), and anti-Gapdh (abcam, ab181602). anti-Dynactin p150 (santa cruz, sc-135890). anti- RILP (Abcam, ab140188)

### Cell culture and cell transfection

HEI-OC1 cells were cultured at 37°C with 10% CO2 in DMEM containing 10% FBS (Pansera, #P30-2602) and 100 IU/ml penicillin (Sigma-Aldrich, A0166). The cells were digested by 0.25% trypsin/EDTA (Life Technologies, #25200056) and then subcultured at 75–80% confluency. When the cells grew to a suitable density, Lipofectamine 2000 Transfection Reagent (Invitrogen, #11668027) was used to transfect plasmids into cells according to the manufacturer’s instructions. The shNC-GFP and shDync1li1-GFP plasmids were generated by OBiO Technology Corp., Ltd. The siNC and siDync1li1 siRNA (5’-CCAGUGCUCGUAGUCUGUATT-3’; 5’-GACAGAGGUGACAGU GUUGTT-3’) were generated by Shanghai GenePharma Company. The shNC-GFP plasmid and siNC siRNA weres used as the negative control, and the shDync1li1-GFP (AGTATGGCGCAGCGCTGATTT) plasmid and siDync1li1 siRNA were used to knock down Dync1li1 in HEI-OC1 cells (House Ear Institute-Organ of Corti 1, a cochlear HC-like cell line). The LC3-RFP plasmid, which was a kind gift from Prof. Zheng Ying (Soochow University, Jiang Su, China) [42–45], was used to label LC3^+^ autophagic vacuoles. The RFP-GFP-LC3 plasmid was purchased from Hanbio Biotechnology (Lot. No. TSB005062-1).

### Data quantification and statistical analysis

The number of myo7a^+^ outer HCs (OHCs) and inner HCs (IHCs) per 100 μm were counted in the apical, middle, and basal turns of the cochlea. For synapse counting, Z-projection was performed to project multiple slides of a Z-stack image onto a single layer, and the numbers of Ctbp2^+^ and PSD95^+^ puncta were counted in the apical, middle, and basal turn of the cochlea per 100 μm using the ImageJ software (S3 Fig). For HEI-OC1 cells, all of the LC3-RFP fluorescent puncta were counted in each HEI-OC 1 cell, and over 50 GFP-shRNA/LC3-RFP double-positive cells were counted. For each experiment, at least three independent experiments were performed. GraphPad Prism 7 software was used to analyze the data and presented as means ± standard errors of the means. A two-tailed, unpaired Student’s t-tests were performed to analyze the data, and p < 0.05 was considered statistically significant.

## Results

### Dync1li1 is expressed in cochlear HCs in both neonatal and adult mice

**Fig 1:**
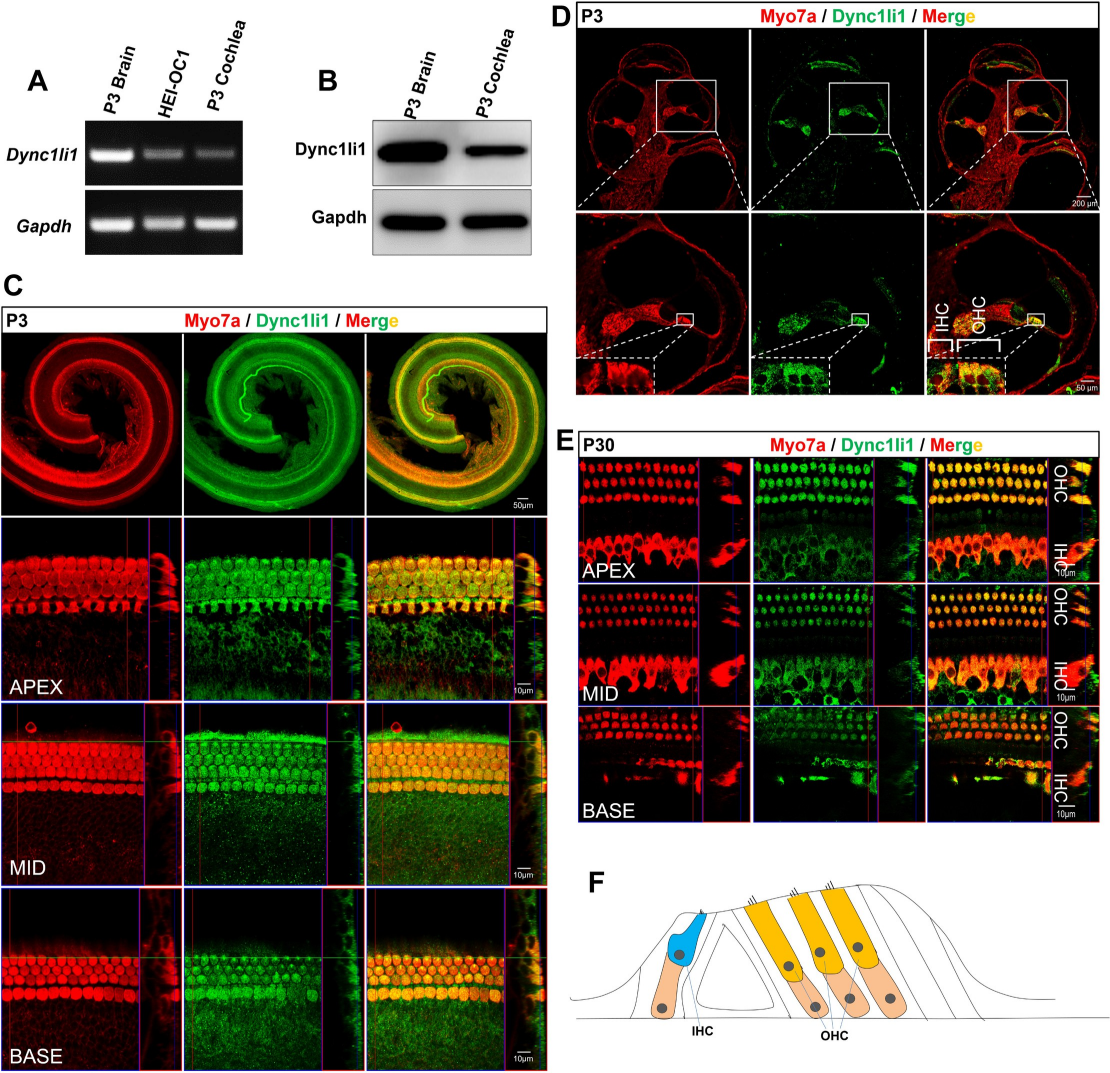
The expression pattern of Dync1li1 in the cochlea of wild-type mice. (A, B) *Dync1li1* mRNA (A) and protein (B) expression in P3 mouse cochlea by RT-PCR and Western blotting, respectively. Brain tissue and the HEI-OC1 cell line were used as the positive controls. (C) Whole mount immunofluorescent staining of Dync1li1 in P3 mouse cochlea. The large square image is a single XY slice, the vertical red line shows the position of the orthogonal slice, which is shown on the right side of each panel, and the blue line on the orthogonal line shows the level of the XY slice on the left. (D) Frozen section immunofluorescent staining of Dync1li1 in P3 mouse cochlea. The white boxes and the dotted lines show enlarged images. (E) Whole mount immunofluorescent staining of Dync1li1 in P30 adult mouse cochlea. For all experiments, scale bars are shown in the figure. (F) A diagram of the IHC and OHC in the cochlea.

RT-PCR showed that *Dync1li1* mRNA was highly expressed in postnatal day 3 (P3) mouse cochlea and in the HEI-OC1 cell line (Fig 1A). Dync1li1 protein was also detected by western blotting in P3 cochlea (Fig 1B). Next, immunofluorescent staining showed that Dync1li1 was highly expressed in HCs in both whole mount and frozen sections of P3 (newborn) and P30 (adult) mouse cochlea. In addition, we also observed that Dync1li1 is also expressed in other types of cells in the organ of Corti, such as spiral neurons and some of supporting cells (Fig 1C–1E). Fig 1F shows a diagram of the IHC and OHC of cochlea. These results suggested that Dync1li1 is highly expressed in HCs and might play important roles in HCs in both the neonatal and adult cochlea.

### *Dync1li1* KO leads to progressive HC loss *in vivo*

**Fig 2:**
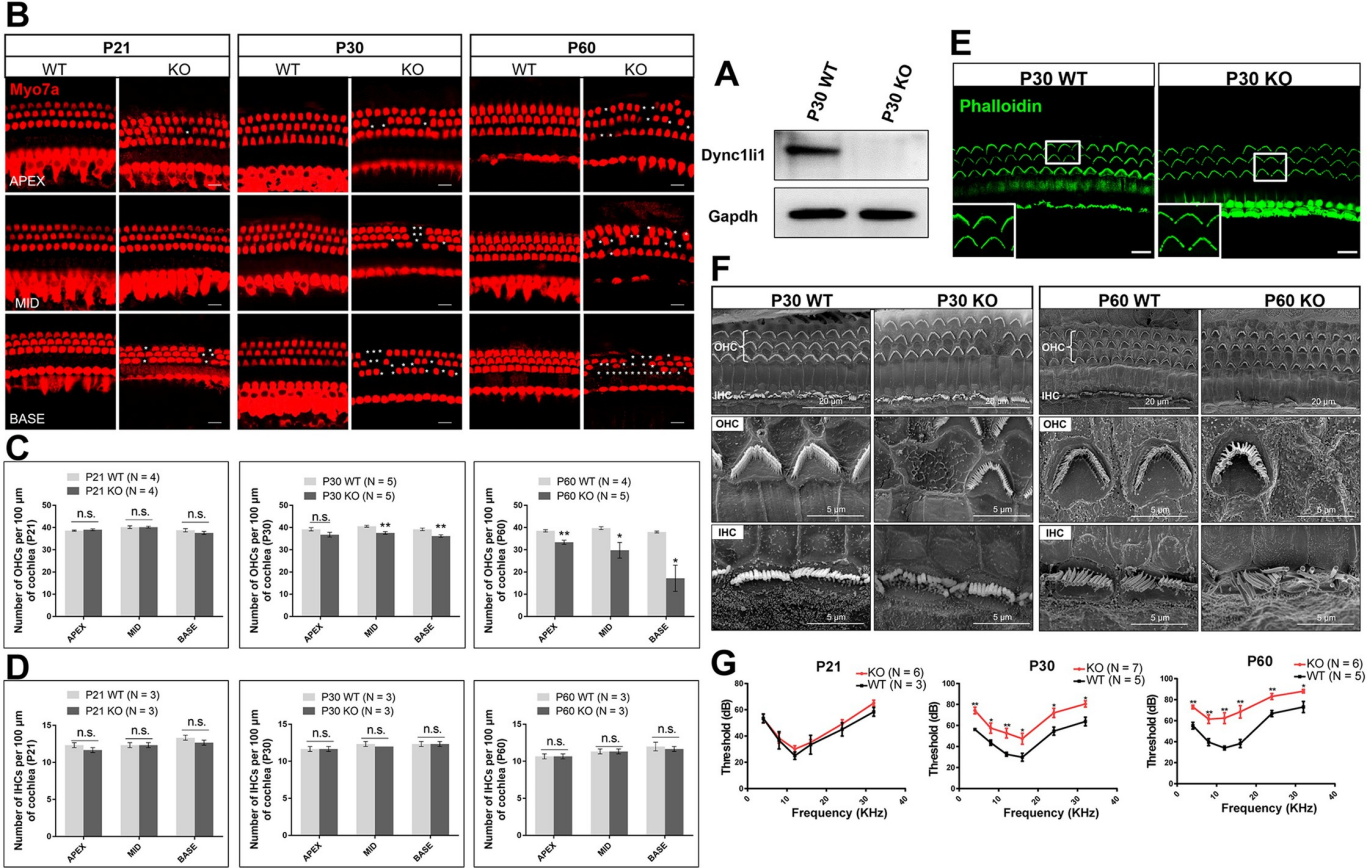
*Dync1li1* KO results in HC loss and hearing loss in adult mice. (A) Western blotting of Dync1li1 in P30 mouse cochlea. Gapdh was used as the internal reference. (B) OHC loss (indicated by asterisks) is seen in the apical (APEX), middle (MID), and basal (BASE) turns of P21, P30, and P60 *Dync1li1* KO and wild-type (WT) mice cochlea. Myo7a (red) was used as the HC marker. (C, D) Quantification of the OHCs (C) and IHCs (D) in the apical, middle, and basal turns of P21, P30, and P60 *Dync1li1* KO and WT mice cochlea. (E, F) Hair bundles were observed by immunofluorescent staining of phalloidin (E) and scanning electron microscopy (F). The enlarged images in the white box in (E) is shown in the lower left corner. (G) The ABR hearing test of *Dync1li1* KO mice and control mice at P21, P30, and P60. For all experiments, scale bars and N number are shown in the figure. *p < 0.05, **p < 0.01, n.s. not significant.

Next, we showed that *Dync1li1* is indeed knocked out in Dync1li1 KO mice (Figs 2A and S4). We first sacrificed mice from neonatal to adult ages to investigate whether HC number is affected by *Dync1li1* KO. We saw no significant HC loss before P21 in *Dync1li1* KO mice, while slight HC loss could be observed from P21 and HC loss gradually became more and more severe as the mice aged (Figs 2B and S1). Quantification of HC loss showed no significant HC loss in P21 mice, although a few HCs were lost in the basal turn. However, significant OHC loss was seen in the apical, middle, and basal turns of the cochlea in P30 and P60 mice (Fig 2C), while the number of IHCs was not significantly changed (Fig 2D). Immunofluorescent staining and scanning electron microscopy (SEM) both showed that the hair bundles of surviving HCs in *Dync1li1* KO mice had normal morphology (Fig 2E and 2F). Because HCs are important sound-sensing cells, we used auditory brainstem response to detect the hearing ability of *Dync1li1* KO mice. Consistent with the degree of HC loss, the hearing thresholds of the *Dync1li1* KO mice were not affected at P21 but were significantly increased at P30 and P60 (Fig 2G). Together these results suggest that Dync1li1 is highly expressed in HCs and that its deletion results in progressive HC loss and hearing loss in adult mice, and thus that Dync1li1 plays important roles in HC survival.

**Fig 3:**
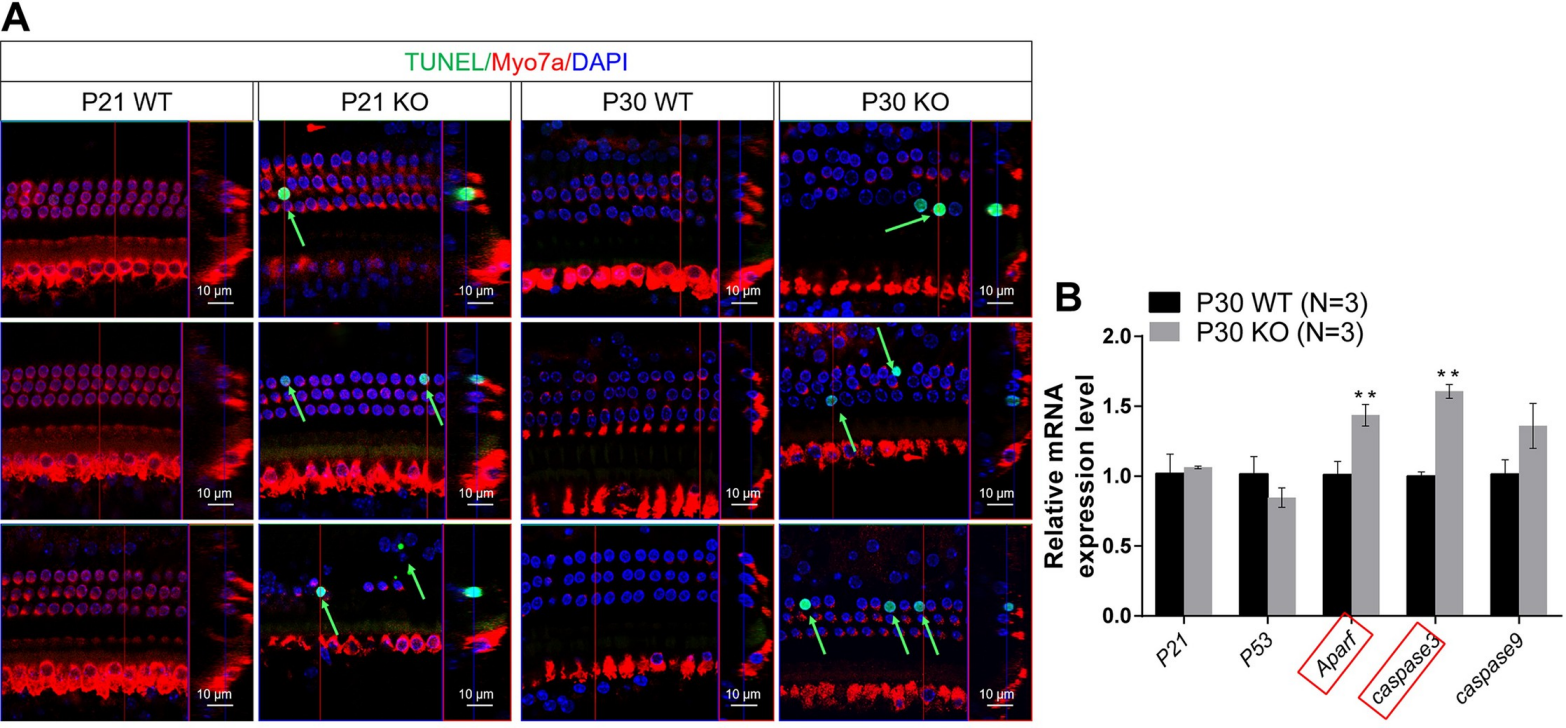
Apoptosis analysis by TUNEL assay in the cochlea of *Dync1li1* KO mice at P21 and P30. (A) TUNEL assay of P21 and P30 *Dync1li1* KO mice and WT control mice. Myo7a and DAPI were used as HC and nuclear markers, respectively. Apoptotic cells are indicated by green arrows. (B) Quantification of the mRNA expression of apoptosis related genes in the cochlea of P30 *Dync1li1* KO mice and the control mice by qPCR. Red boxes indicate the genes with significant expression differences. For all experiments, scale bars and N number are shown in the figure. **p < 0.01.

TUNEL signals, which are indicative of apoptosis, were observed in HCs of both P21 and P30 *Dync1li1* KO mice, but not in the control group (Fig 3A). Real-time qPCR results also showed that the expression of apoptosis-related genes, such as *Aparf* and *Caspase3*, were significantly upregulated in the *Dync1li* KO mouse cochlea (Fig 3B). Together, these results indicate that HC loss in *Dync1li1* KO mice was due to HC apoptosis.

### Dync1li1 KO decrease the stability of Dynein complex in HCs

**Fig 4:**
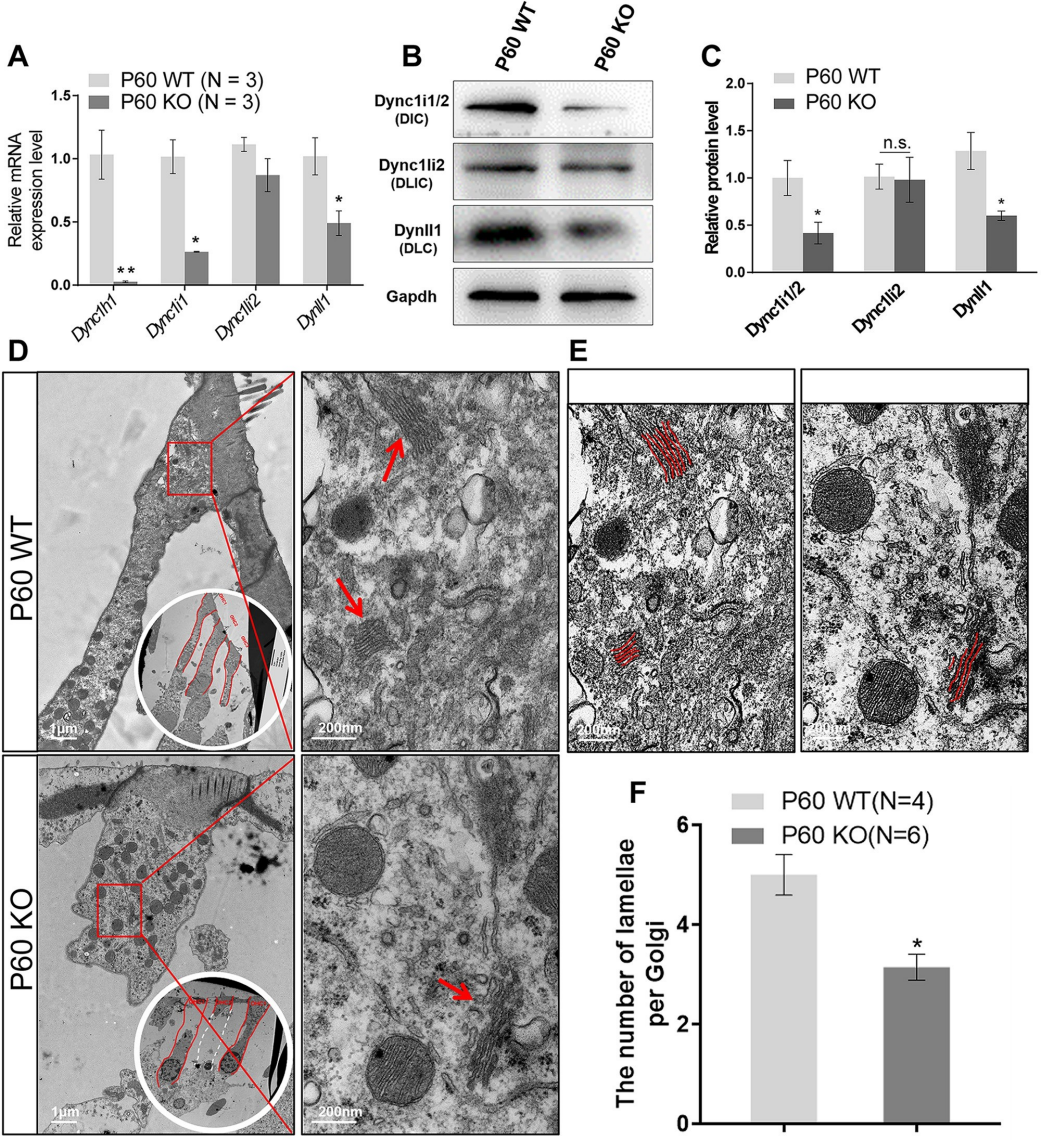
Dync1li1 deficiency affect the integrity of Dynein complex and Golgi apparatus. (A) Quantification of the mRNA expression of important subunits in dynein complex (*Dync1h1*, *Dync1i1*, and *Dynll1*) in P60 *Dync1li1* KO and the control mice by qPCR. N = 3. N refers to 3 independent qPCR experiments were performed. (B, C) Western blotting (B) and quantification of the western blotting (C) of the Dynein subunit in the cochlea of P60 *Dync1li1* KO mice. Gapdh was used as the internal reference. N is indicated in the figure. (D, E) TEM of OHCs in P60 *Dync1li1* KO and control mice. The Golgi apparatus is indicated by red arrows in (D) and red lines in (E). (F) Quantification of the number of lamellae per Golgi. For all experiments, scale bars are shown on the figure and N is indicated in the figure. *p < 0.05, ***p < 0.001.

Because Dync1li1 is an important part of the dynein complex, which is crucial in all eukaryotic cells for transporting a variety of essential cargoes toward the minus end of microtubules (also called retrograde transport), we speculated that HC apoptosis caused by *Dync1li1* KO might be related to impaired retrograde transportation. Thus, we first detected the expression of other components of the dynein complex to determine the stability of dynein. The mRNA levels of *Dync1h1* (DHC), *Dync1i1* (one of the DIC genes), and *Dync1l1* (one of the DLC genes) were all significantly downregulated in P60 *Dync1li1* KO mouse cochlea (Fig 4A), and the protein level of Dync1i1/2 (DIC) and Dyncll1 (DLC) were also significantly downregulated, with Dync1i1/2 being the most pronounced and the expression of Dync1li2 was not significantly changed in P30 *Dync1li1* KO mice (Fig 4B and 4C). We then used TEM to explore the effects on transport-related organelles in cochlear HCs, such as the structure of the endoplasmic reticulum (ER) and the Golgi apparatus, and we found that the number of lamellae per Golgi was significantly reduced in *Dync1li1* KO OHCs compared to the control group and that the Golgi apparatus was thinner in the Dync1li1 KO OHCs (Fig 4D–4F). Together, these results indicate that deletion of Dync1li1 leads to unstable dynein complexes and to decreased stacks of Golgi apparatus in cochlear OHCs.

**Fig 5:**
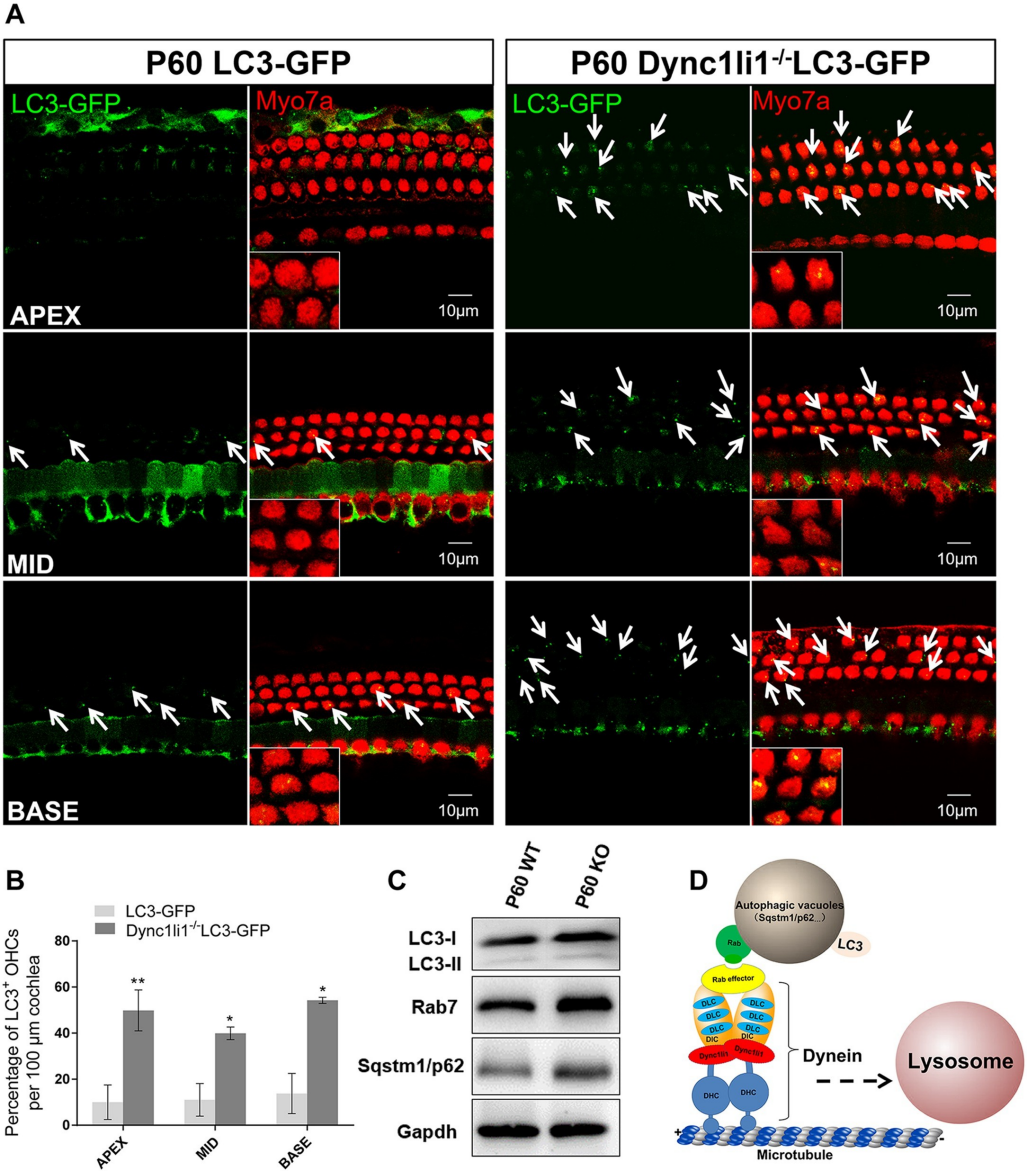
*Dync1li1* deficiency induced the accumulation of autophagosomes vacuoles in HCs. (A) Immunofluorescence of LC3 (green puncta, indicated by white arrows) in HCs of P60 Dync1li1^−/−^LC3-GFP mice and control mice. Myo7a was used as HC marker. The enlarged images are shown in the lower left corner. (B) Quantification of the number of LC3^+^ OHCs. N = 3. (C) Western blotting of the LC3 (LC3-I, 16 kDa; LC3-II, 14 kDa.), Rab7, and Sqstm1/p62 in the cochlea. Gapdh was used as the internal reference. (D) Schematic of the role of dynein in mediating autophagosome–lysosome fusion. Rab links autophagosomes to dynein to mediate microtubule-dependent minus-end-directed transportation towards the lysosome. For all experiments, scale bars are shown in the figure. *p < 0.05, **p < 0.01.

HC apoptosis caused by Dync1li1 KO is due to impaired transportation of autophagosomes to lysosomes Considering that dynein plays crucial roles during the autophagic process (Fig 5D) and that autophagy is involved in apoptosis [46], we hypothesized that the HC apoptosis caused by Dync1li1 KO is due to impaired transportation of autophagosomes to lysosomes. To test this, we first detected the LC3 signal (autophagic vacuoles marker) in HCs of P60 Dync1l*i1*^−/−^LC3-GFP mice and found significantly more LC3-positive puncta in the OHCs of *Dync1li1*^−/−^LC3-GFP mice compared to the LC3-GFP–only control mice (Fig 5A and 5B). Western blotting showed that the LC3 and Sqstm1/p62 protein level were upregulated in P60 *Dync1li1* KO mouse cochlea, suggesting that autophagic vacuoles were aggregated in the HCs (Fig 5C). Moreover, Rab7, an adaptor protein during the maturation of autolysosomes [47,48], was also upregulated in the cochlea of P60 *Dync1li1* KO mice (Fig 5C), which indicated that newly formed autophagosomes (with Rab7 on their surface) could not be transported to lysosomes for degradation and thus had accumulated in the HCs.

**Fig 6:**
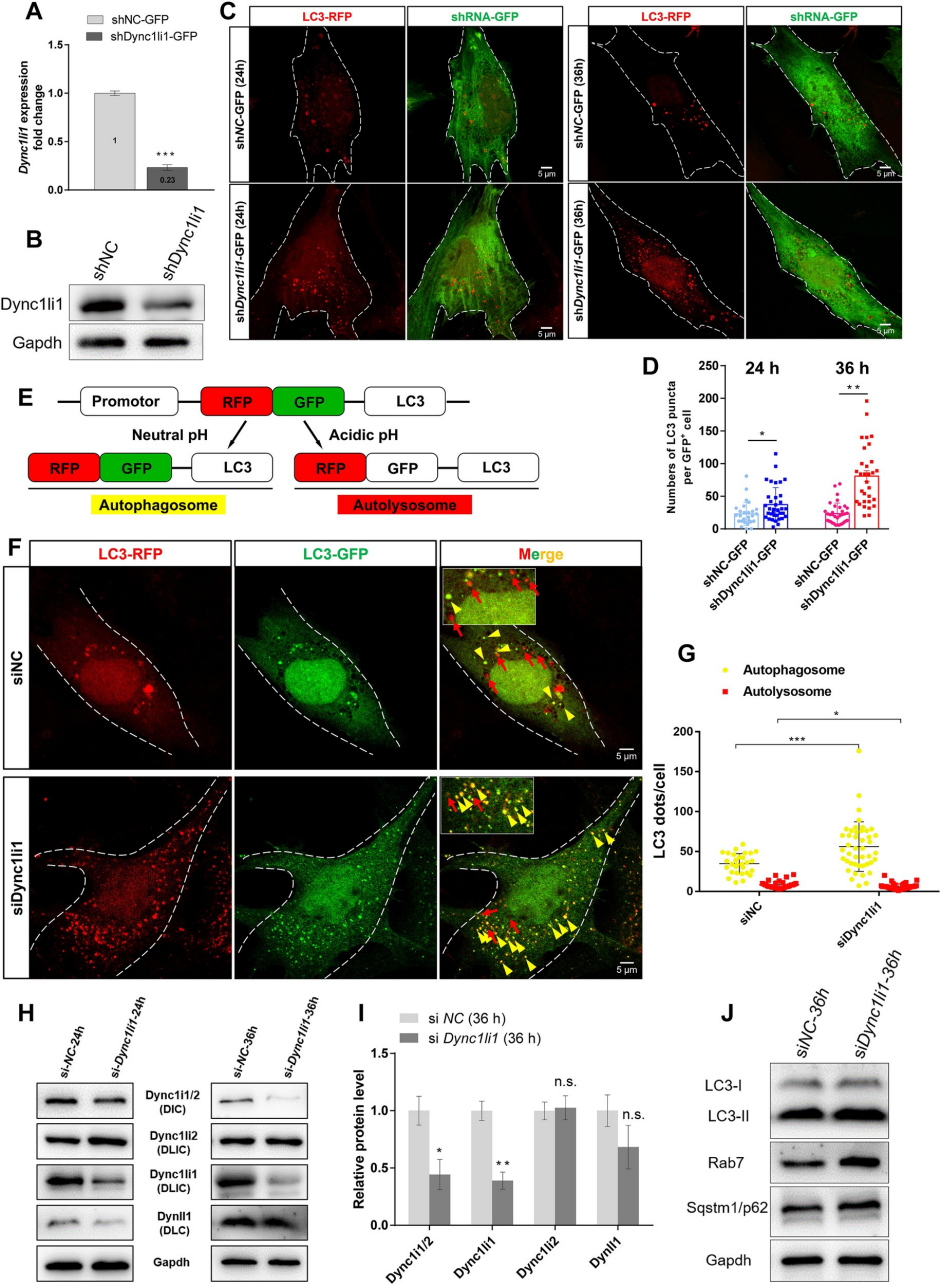
Accumulation of autophagosomes in *Dync1li1* KD HEI-OC1 cells. (A, B) OC1 Cells were transfected with sh*Dync1li1-*GFP shRNA, and qPCR analysis (A) and Western blotting (B) were used to test the knockdown efficiency of Dync1li1. sh*NC-*GFP was used as control shRNA. The cells were harvest after 36h transfection. (C, D) sh*NC-*GFP and sh*Dync1li1-*GFP were cotransfected with LC3-RFP plasmids into OC1 cells for 24 h and 36 h, respectively. Red dots in (C) indicate the LC3 puncta (autophagic vacuoles), which were quantified in (D). (E) Schematic of the working principle of the RFP-GFP-LC3 plasmid. In a neutral environment (autophagosome), LC3 is expressed with both GFP and RFP fluorescencent proteins, and thus the autophagosome dots (RFP^+^GFP^+^) are yellow. In an acidic environment (autolysosome), GFP fluorescence is quenched, and thus the autolysosome dots (RFP^+^) are red. (F, G) siNC and siDync1li1 siRNA were cotransfected with RFP-GFP-LC3 plasmid for 36 h. Yellow arrow heads and red arrows in (F) indicate the autophagosomes (yellow dots) and the autolysosomes (red dots), respectively. The enlarged images are shown in the upper left in (F). The number of autophagosomes and autolysosomes in the *Dync1li1* KD cells and the control cells were quantified in (G). (H, I) siNC and siDync1li1 siRNA were transfected for 24 h and 36 h, respectively. Western blotting (H) and quantification of Dynein subunit proteins after siRNA transfection. Quantification of protein expression levels at 36 h after siRNA transfection (I). (J) Western blotting of LC3, Sqstm1/p62 and Rab7 at 36 h after siRNA transfection. For all experiments, scale bars are shown in the figure. *p < 0.05, **p < 0.01, n.s. not significant.

Next, we verified these results in the HC-like HEI-OC1 cell line [49]. We used shRNA to knock down *Dync1li1* in the HEI-OC1 cell line and confirmed the KD efficiency (Fig 6A and 6B). We then transfected the LC3-RFP plasmid into HEI-OC1 cells and quantified the LC3 puncta, and we found that the number of LC3 puncta was significantly increased in the *Dync1li1* KD group at both 24 h and 36 h after transfection compared to the controls (Fig 6C and 6D).

RFP-GFP-LC3 is a tool plasmid for detecting the level of autophagy in cells as illustrated in Fig 6E [50]. There are two fluorescent protein, red RFP and green GFP, expressed as a fusion protein with LC3, in which GFP is a pH sensitive protein. When in the autophagic vacuoles with a neutral pH (autophagosome), RFP and GFP both show fluorescent signal, and thus the LC3 dots are yellow (GFP+/RFP+). When in the autophagic vacuoles with an acidic pH (autolysosome), GFP cannot show fluorescent signal and only RFP can show red fluorescent signal, and thus the LC3 dots are red. Therefore, we can use this plasmid to measure autophagic flux in the *Dync1li1* KD group and the control group. And we found that in *Dync1li1* KD OC1 cells, the number of autophagosomes (GFP+/RFP+) were significantly more than that in the control group, while the number of autolysosomes (GFP-/RFP+) was less than that in the control group (Fig 6F and 6G). These data suggested that *Dync1li1* KD led to accumulation of autophagosomes which cannot be eliminated by transporting to lysosomes to form autolysosomes. Moreover, we also found that the protein level of Dync1i1/2 (DIC) and Dyncll1 (DLC) were also significantly down regulated, the expression of Dync1li2 was not changed, and the protein level of LC3 and Sqstm1/p62 were up regulated in *Dync1li1* KD group (Fig 6H and 6I). These results are consistent with the changes in protein level in *Dync1li1* KO mouse cochlea. Therefore, we conclude that knock down of *Dync1li1 i*n vitro led to impaired transportation of autophagosomes to lysosomes and to abnormal accumulation of autophagosomes in HC-like HEI-OC1 cells.

**Fig 7:**
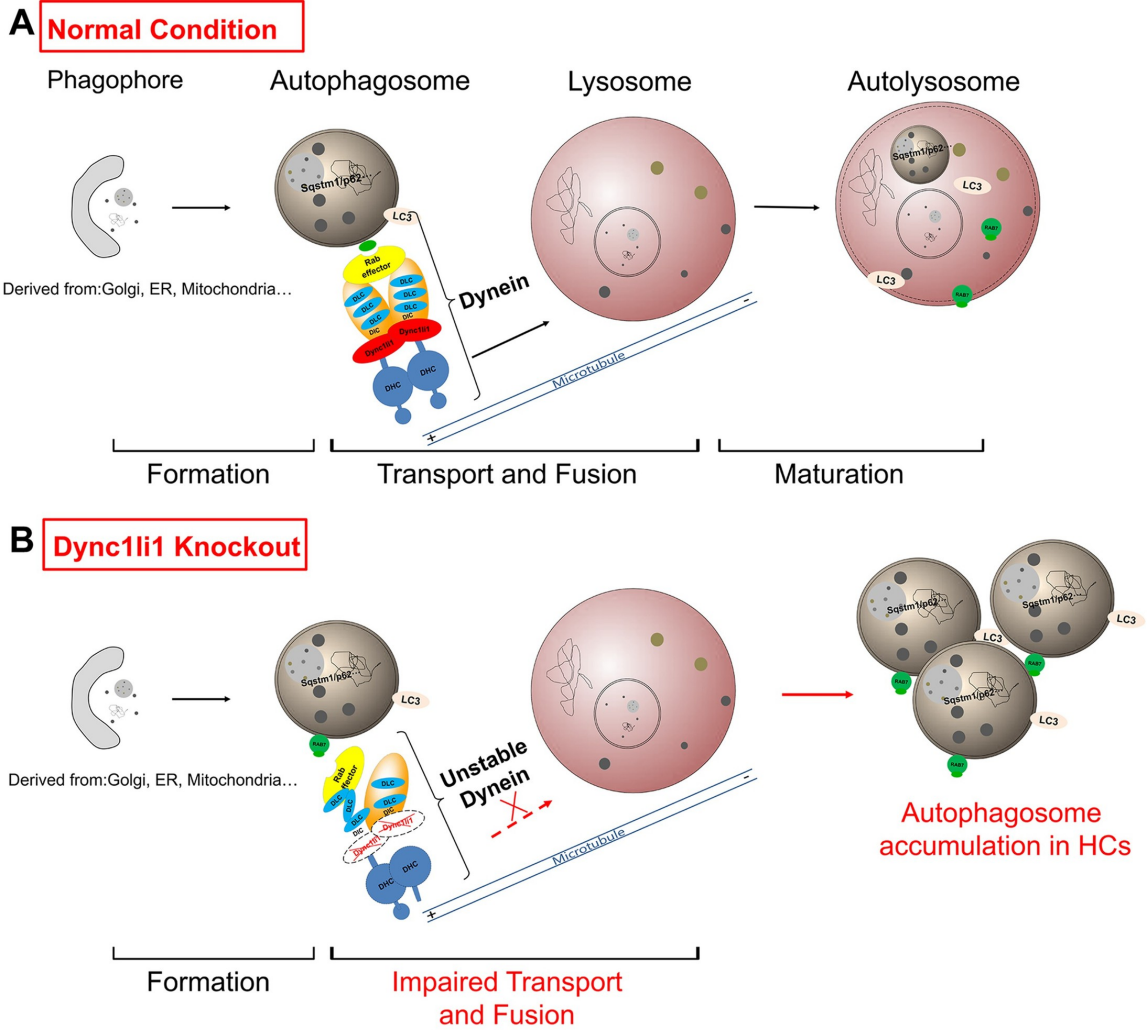
Working model of dynein-dependent autolysosome clearance in cochlear HCs. (A) Under normal conditions, phagophores (derived from the ER, Golgi, mitochondria, etc.) expand and form late autophagosomes, which bind to the dynein complex through Rab7. Late autophagosomes, with both LC3 and Rab7 expressed on their membranes, are transported by dynein along microtubules to lysosomes to form autolysosomes and to be digested. This autophagic flux maintains homeostasis in HCs. (B) When *Dync1li1* is knocked out in cochlear HCs, the fusion of late autophagosomes with lysosomes is impaired by the unstable dynein complex. Therefore, large numbers of late autophagosomes are accumulated in the HCs, which disrupts normal autophagic flux and leads to HC apoptosis.

In summary, the mechanisms we identified are shown in Fig 7. Under normal conditions, late autophagosomes with harmful substances produced in HCs are transported by dynein to be fused with lysosomes and form autolysosomes for subsequent degradation, thereby maintaining cell homeostasis. When Dync1li1 is defective or missing, the dynein complex becomes unstable and cannot effectively transport late autophagosomes to lysosome for degradation, which leads to the accumulation of these autophagosomes with harmful substances. This disruption in cell homeostasis triggers HC apoptosis and thus leads to hearing loss.

## Discussion

Dynein plays critical roles in the central nervous system through its effects on nuclear migration and retrograde transport of various cargos [51]. In this study, we found that one member of the dynein complex, Dync1li1, was highly expressed in cochlear HCs, and *Dync1li1* KO mice showed progressive hearing loss along with early onset of HC apoptosis. Further study revealed that Dync1li1 deletion decreased the expression level of other members of dynein complex, including the DHC, DIC, and DLC, which further reduced the number of lamellae in the Golgi apparatus and led to the accumulation of autophagosomes in HCs. Together our data suggest that the unstable dynein complex caused by *Dync1li1* KO affects Golgi-related transport and the transport of autophagosomes to lysosomes and that this ultimately results in HC apoptosis in the cochlea.

The cilia of HCs (called HC bundles) show a highly ordered arrangement that makes them highly sensitive to vibration of the fluid environment, and the planar cell polarity (PCP) of HC bundles is essential for hearing function [52–55]. It is reported that Dync1li1 knockout mice show deficient ciliogenesis of photoreceptors [20]. Here we found that Dync1li1 is highly expressed in auditory HC cytoplasm, rather than in HC bundles, in both neonatal and adult mouse cochlea. Dync1li1 KO mice showed progressive HC loss and hearing loss at all frequencies, but no changes were observed in the morphology or PCP of HC bundles. These results indicated that the function of Dync1li1 in HCs was not involved in the formation or PCP of the hair bundles.

The TUNEL assay showed that HC loss caused by Dync1li1 deficiency is due to early onset of HC apoptosis. Previous study showed that separation of DLIC from the dynein complex result in unstable DHC in vertebrate [7]. In the mouse retina, loss of Dync1li1 reduced the protein levels of DHC、DIC and DLC and therefore impair the transport ability of dynein [20]. Consistent with previous reports, here we also found that deletion of Dync1li1 led to decreased expression of the DHC, DIC, and DLC in mouse cochlea and thus caused the destabilization of the dynein complex in cochlear HCs. In the photoreceptor cells, LIC1 deletion also increase dynactin P150 (a dynein adaptor linking dynein to cargoes) protein expression [20], we also investigate the protein level of P150 in the KO Dync1lil mouse cochlea (S2C Fig).

Considering that dynein is involved in maintaining the architecture of the ERGIC (ER-Golgi intermediate compartment) and that inhibition of dynein leads to fragmentation and dispersion of the Golgi apparatus [15,56–58], we also investigated the effects of *Dync1li1* deletion on the morphology of the ER and Golgi apparatus in HCs. Our TEM data showed that the lamellae of the Golgi apparatus were thinner in *Dync1li1* KO OHCs, which suggested that *Dync1li1* deletion led to disorganization of transport processes in the Golgi apparatus. We also measured the ER stress level by measuring the protein level of Calnexin and P-eIF2α, and we found that neither of them were significantly changed (S2A and S2B Fig). Since RILP is also an important adapter for dynein, we investigated the expression of RILP in the KO Dync1lil mouse cochlea. Interestingly, we found that the protein level of RILP was not significantly changed (S2D Fig).We also investigated the location and the number of auditory ribbon synapses in *Dync1li1* KO mice, but we did not observe any abnormalities. Both the location and the number of ribbon synapses were normal in HCs of *Dync1li1* KO mice (S3 Fig), which indicated that *Dync1li1* deficiency did not affect the formation of ribbon synapses.

Accumulating studies have shown that the dynein complex is involved in autophagic processes and dynein-dependent retrograde transport of autophagic vacuoles is essential for the survival of neurons [37,38,59–61]. Dynein inhibition impair the process of clearance of aggregate-prone proteins in the drosophila and mouse model of Huntington’s disease [35]. The mutation of dynein weaken the clearance of mutant huntingtin fragments by cross the HdhHD mice (mouse model of Huntington disease [62]) with Dnchc1Loa (ethylnitrosourea-induced missense mutation in the dynein heavy chain 1[63]), and the level of LC3- II expression in Hdh+/+ Dnchc1Loa/+ mice was increased [35].

Basal autophagy is important for homeostasis in postmitotic cells [64,65]. In the inner ear, basal autophagic flux can be detected in cochlear HCs and is essential for hearing in mice [66,67], and autophagy-deficient mice show impaired biogenesis of otoconia [68]. However, there is no research about the relation between dynein and autophagy in cochlear HCs. In our study, we observed that the endogenous LC3 puncta were significantly increased in both HCs and HC-like HEI-OC1 cells after *Dync1li1* KO or KD, which suggested that without dynein the process of autophagosome clearance was abnormal and that this led to the accumulation of autophagosomes in HCs that in turn triggered apoptosis in HCs and subsequent hearing loss.

Many studies have shown that Rab7 is required for the complete autophagic flux and that it regulates the process of autophagosome-lysosome fusion [69,70]. These studies indicated the strong interaction between dynein and Rab7 in the process of late autophagosome-lysosome fusion. Here, our results showed that both the protein level of Rab7 and LC3 were significantly increased in cochlear HCs of *Dync1li1* KO mice and *Dync1li1* KD HEI-OC1 cells *in vitro*, which suggested that late autophagosomes accumulated in *Dync1li1*-deficient cells. Therefore, we speculated that deletion of *Dync1li1* impaired the transportation of late autophagosomes to lysosomes, such that LC3+ autophagosomes could not be cleared and therefore accumulated in the HCs. However, it is worth noting that Rab7 is also a marker of late endosomes [71] and that Rab7 is required for mitophagosome formation by regulating phagophore transport [72,73]. Therefore, in future work we will further explore the effects of *Dync1li1* deletion on other Rab7-mediated transport processes in the mouse cochlea.

In summary, we have identified new roles for Dync1li1 in maintaining the survival of mouse cochlear HCs. We show that *Dync1li1* KO leads to destabilization of the dynein complex and that this results in impaired transport of late autophagosomes to lysosomes. Therefore, LC3^+^ autophagosomes cannot be cleared and thus accumulate in HCs, which leads to HC apoptosis and hearing loss in adult mice.

## Supporting information

S1 Fig No HC loss is observed in the cochleae of *Dync1li1* KO mice at P1 and P14. (A, B) Immunofluorescent staining of Myo7a and Phalloidin in P1 (A) and P14 (B) *Dync1li1* KO mice and WT control mice cochleae, respectively. Myo7a was used as HC marker. Phalloidin was used as HC bundle marker. (C, D) Quantification of OHC (C) and IHC (D) number in the apical (APEX), middle (MID), and basal (BASE) turns of P1 *Dync1li1* KO and control mice cochleae. (E, F) Quantification of OHC (E) and IHC (F) number in the apical (APEX), middle (MID), and basal (BASE) turns of P14 *Dync1li1* KO and control mice cochleae. For all experiments, scale bar are shown on the figure, N is indicated in the figure, n.s. not significant.
(TIF)

S2 Fig The analysis protein level of expression of ER stress-related proteins, P150 and RILP in Dync1li1 KO mice at P30. (A, B,C,D) Western blotting of Calnexin (A), P-eIF2α (B), P150 (C) and RILP (D) in the cochleae of P30 *Dync1li1* KO mice and WT mice. Calnexin was used as the ER marker and P-eIF2α was used as the ER stress marker. Gapdh was used as the internal control.
(TIF)

S3 Fig The ribbon synapses of IHCs in P21 *Dync1li1* KO mice. (A) Immunofluorescent staining of Ctbp2 and PSD95 in P21 *Dync1li1* KO mice and WT control mice IHCs, respectively. Images were taken from the MID turn of the cochleae. The enlarged image in the white box is shown in the lower right corner. Ctbp2 was used as presynaptic marker. PSD95 was used as postsynaptic marker. (B) Quantification of the number of synapese from MID turn of *Dync1li1* KO and WT mice. For all experiments, scale bars are shown on the figure, n.s. not significant.
(TIF)

S4 Fig The full blot of LIC in Fig 2A. (TIF)

S1 Table Primers for real-time qPCR detection and relative quantification of gene expression in mouse. (DOCX)

S1 Note Abbreviations. (DOCX)
