# Supplementary figures and images for: Dync1li1 is required for the survival of mammalian cochlear hair cells by regulating the transportation of autophagosomes
Source: PLoS Genet. 2022 Jun 21;18(6):e1010232. doi: 10.1371/journal.pgen.1010232 (PMC9249241; doi:10.1371/journal.pgen.1010232)

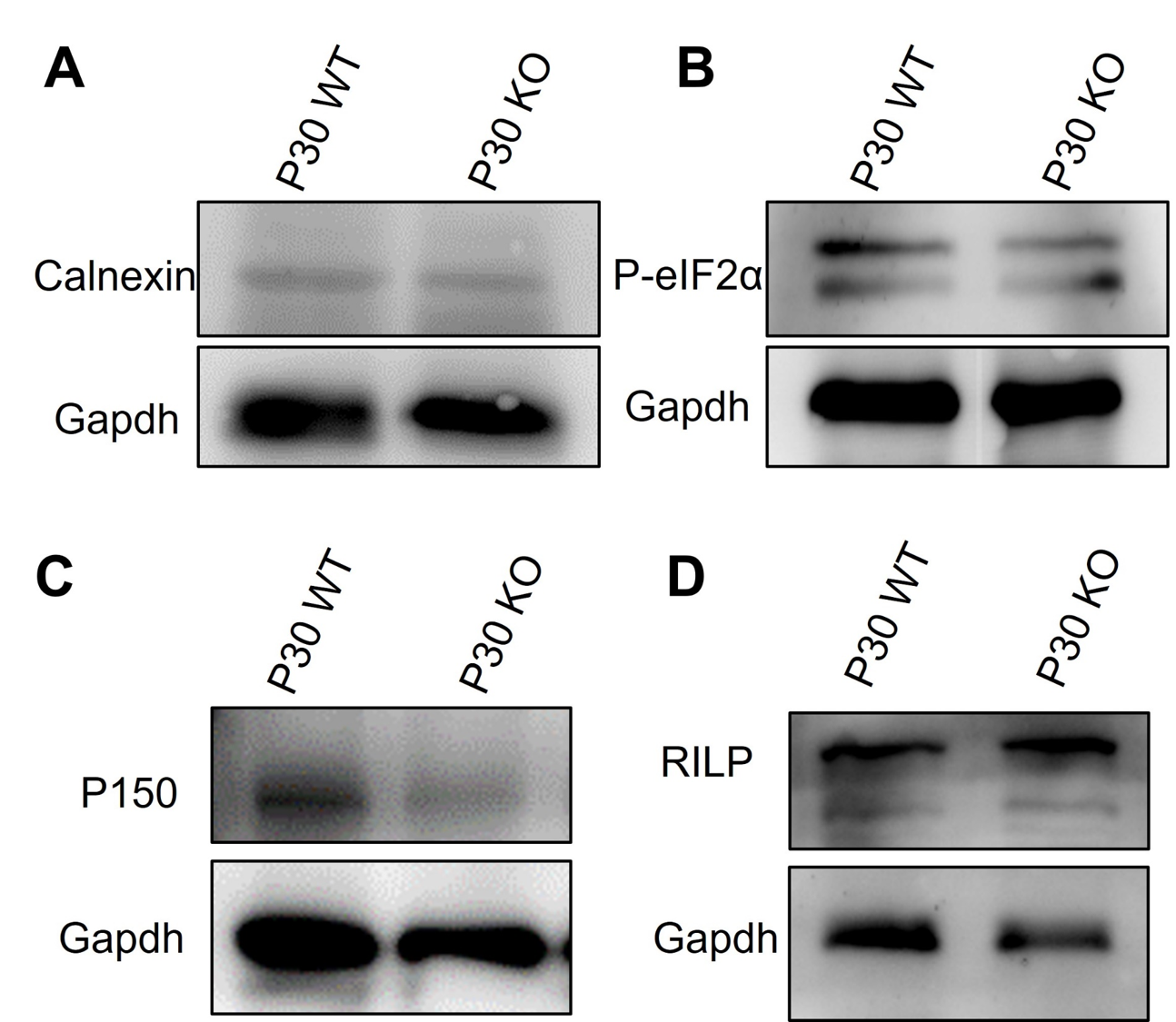

Supplement: S2 Fig — (A, B,C,D) Western blotting of Calnexin (A), P-eIF2α (B), P150 (C) and RILP (D) in the cochleae of P30 Dync1li1 KO mice and WT mice. Calnexin was used as the ER marker and P-eIF2α was used as the ER stress marker. Gapdh was used as the internal control. (TIF) [file pgen.1010232.s002.tif]

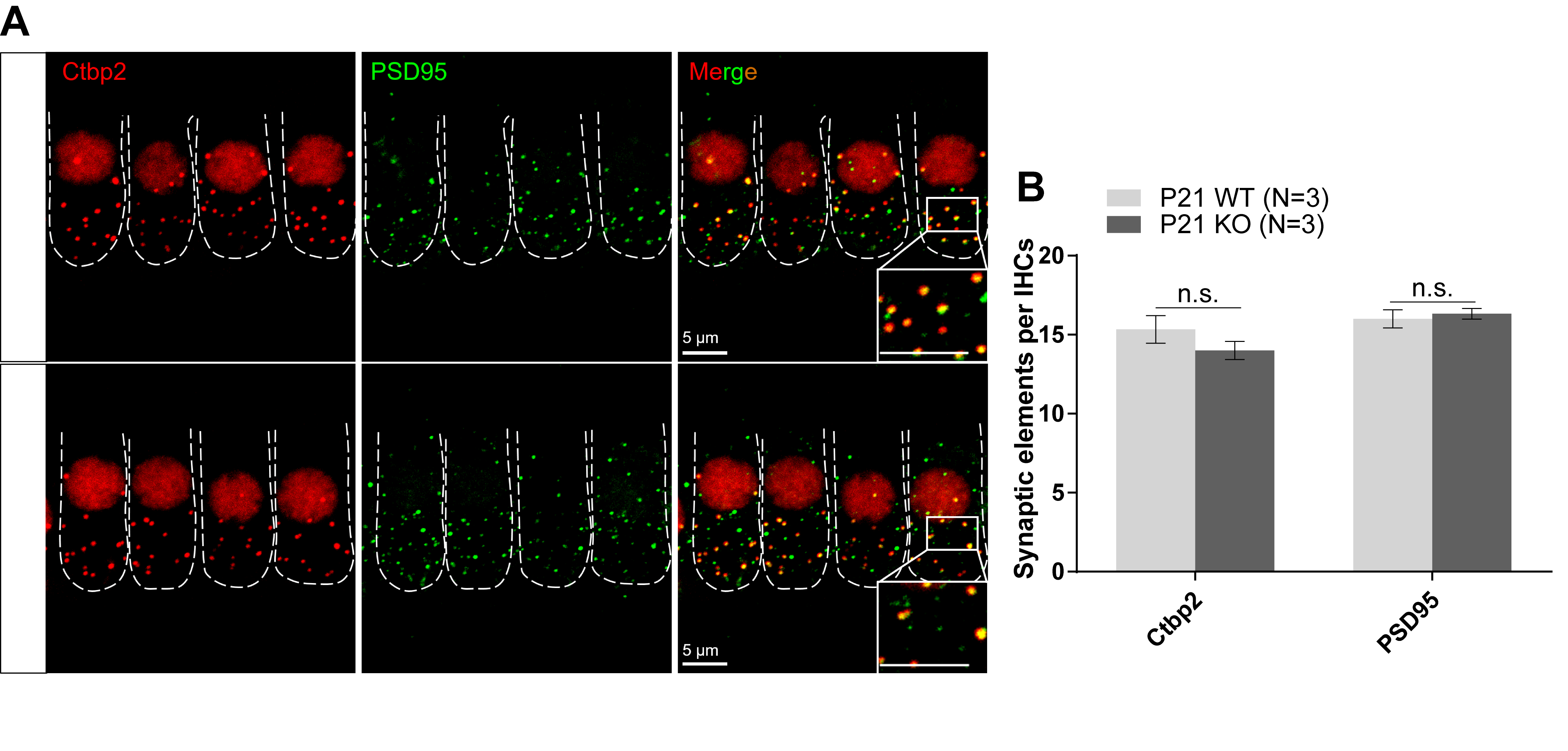

Supplement: S3 Fig — (A) Immunofluorescent staining of Ctbp2 and PSD95 in P21 Dync1li1 KO mice and WT control mice IHCs, respectively. Images were taken from the MID turn of the cochleae. The enlarged image in the white box is shown in the lower right corner. Ctbp2 was used as presynaptic marker. PSD95 was used as postsynaptic marker. (B) Quantification of the number of synapese from MID turn of Dync1li1 KO and WT mice. For all experiments, scale bars are shown on the figure, n.s. not significant. (TIF) [file pgen.1010232.s003.tif]
