# Supplementary material for: Dync1li1 is required for the survival of mammalian cochlear hair cells by regulating the transportation of autophagosomes
Source: PLoS Genet. 2022 Jun 21;18(6):e1010232. doi: 10.1371/journal.pgen.1010232 (PMC9249241; doi:10.1371/journal.pgen.1010232)
Supplement: S1 Table — (DOCX) [file pgen.1010232.s005.docx]

**S1 Table**

Primers for real-time qPCR detection and relative quantification of gene expression in mouse

| Gene name | primer | Real-time qPCR primer sequence (5'–3') |
| --- | --- | --- |
| *Gapdh* | Forward | AGGTCGGTGTGAACGGATTTG |
|  | Reverse | TGTAGACCATGTAGTTGAGGTCA |
| *Dync1li1* | Forward | CCATGAGCGCATCGCAATC |
|  | Reverse | AGTAGCCCTTTGTGGTACAGA |
| *Dync1h1* | Forward | AAGCACCTGCGTAAGCTGG |
|  | Reverse | GCGGGTCTGACAGGAACTTG |
| *Dync1i1* | Forward | AGCCCGTTCAAGATGACTCC |
|  | Reverse | GTGCAGTCGTCTCCCAAGTT |
| *Dynll1* | Forward | ATTGCGGCCCATATCAAGAAG |
|  | Reverse | GTGCCACATAACTACCGAAGTTT |
| *P21* | Forward | CCTGGTGATGTCCGACCTG |
|  | Reverse | CCATGAGCGCATCGCAATC |
| *P53* | Forward | CTCTCCCCCGCAAAAGAAAAA |
|  | Reverse | CGGAACATCTCGAAGCGTTTA |
| *Aparf1* | Forward | AGTGGCAAGGACACAGATGG |
|  | Reverse | GGCTTCCGCAGCTAACACA |
| *Caspase 3* | Forward | TGGTGATGAAGGGGTCATTTATG |
|  | Reverse | TTCGGCTTTCCAGTCAGACTC |
| *Caspase 9* | Forward | TCCTGGTACATCGAGACCTTG |
|  | Reverse | AAGTCCCTTTCGCAGAAACAG |
