# Supplementary material for: Dync1li1 is required for the survival of mammalian cochlear hair cells by regulating the transportation of autophagosomes
Source: PLoS Genet. 2022 Jun 21;18(6):e1010232. doi: 10.1371/journal.pgen.1010232 (PMC9249241; doi:10.1371/journal.pgen.1010232)
Supplement: S1 Note — (DOCX) [file pgen.1010232.s006.docx]

**S1 Note**

**Abbreviations**

ABR: auditory brainstem response

Bax: Bcl2-associated X protein

Bcl2: B-cell leukemia/lymphoma 2

DHC: Cytoplasmic dynein 1 heavy chain

DIC: Cytoplasmic dynein 1 intermediate chain

DLIC: Cytoplasmic dynein 1 light intermediate chain

DLC: Cytoplasmic dynein 1 light chain

HCs: hair cells

MAP1LC3/LC3: microtubule-associated protein 1 light chain 3

Myo7a: myosin VII A

qPCR: quantitative real-time PCR

SEM: Scanning electron microscope

TEM: Transmission electron microscope

TUNEL: terminal deoxynucleotidyl transferase dUTP

PCP: planar cell polarity

SEM：Scanning Electron Microscopy

TEM：Transmission Electron Microscopy
